# Metabolomic profiling of indigenous lactic acid bacteria reveals functional traits shaping the flavor and bioactivity of Ecuadorian coffee

**DOI:** 10.3389/fmicb.2025.1697280

**Published:** 2025-11-19

**Authors:** Victor Cifuentes, Ioana C. Marinas, George Cătălin Marinescu, Roua Gabriela Popescu, Mariana C. Chifiriuc, Gabriela N. Tenea

**Affiliations:** 1 Biofood and Nutraceutics Research and Development Group, Faculty of Engineering in Agricultural and Environmental Sciences, Universidad Técnica del Norte, Ibarra, Ecuador; 2 Research Institute of the University of Bucharest-ICUB, University of Bucharest, Bucharest, Romania; 3 Independent Research Association, Bucharest, Romania; 4 Blue Screen SRL, Bucharest, Romania

**Keywords:** lactic acid bacteria, metabolites, *Coffea arabica*, LC-MS/MS untargeted metabolomics, harmane, polyphenols

## Abstract

**Introduction:**

Microbial fermentation by lactic acid bacteria (LAB) significantly influences the flavor, quality, and functional attributes of coffee. However, the specific metabolic outputs and roles of LAB native strains to distinct *Coffea arabica* ecosystems remain insufficiently understood. This study aimed to characterize the metabolite profiles and functional signatures of cell-free supernatants (CFS) from six indigenous LAB strains isolated from three Ecuadorian coffee varieties, *C. arabica* var. Typica (TYP), *C. arabica* var. Yellow Caturra (CATY), and *C. arabica* var. Red Caturra (CATR), harvested at two ripening stages (green and yellow/red).

**Methods:**

Metabolite profiling was performed using capillary liquid chromatography tandem mass spectrometry (LC-MS/MS) with a SWATH-based data-independent acquisition (DIA) strategy in positive electrospray ionization (ESI+) mode, enabling detection of metabolites associated with flavor development, stress response, and antimicrobial potential. Functional group analysis via attenuated total reflectance Fourier transforms infrared (ATR-FTIR) spectroscopy provided insights into structural and biochemical changes, including protein, carbohydrate, and lipid modifications during LAB activity. Total polyphenol content (TPC) and total flavonoid content (TFC) were quantified to assess nutritional and antioxidant shifts.

**Results:**

Strain-specific metabolic signatures were identified. *Lactiplantibacillus* strains (B3, B6, B9, B10, B17) showed enriched biosynthesis of harmala alkaloids, isoflavonoids, indole derivatives, and bioactive peptides (e.g., FruLeuIle), which may contribute to enhanced aroma and bioactivity. *Weissella* (B19) exhibited a simpler profile, dominated by organic acids and benzene derivatives, potentially enhancing acidity and freshness. FTIR analysis revealed that B6, B10, B17, and B19 released distinctive extracellular polysaccharides, proteins, and aromatic compounds, shaping the fermented matrix.

**Conclusion:**

This study demonstrates the functional diversity of indigenous LAB strains from *C. arabica* cherries, showing that their strain-specific metabolic signatures reshape the fermentation matrix and highlighting their potential for targeted microbial selection to enhance flavor complexity, quality, and the market value of Ecuadorian specialty coffees.

## 1 Introduction

*Coffea arabica L*., which represents around 70% of global coffee consumption, owes its distinctive flavor, aroma, and sensory quality not only to its genetic background and agronomic conditions but also to the complex microbial interactions that occur during post-harvest fermentation (Todhanakasem et al., 2024; Shen et al., 2025). Microbial communities comprising bacteria, yeasts, and filamentous fungi drive the biochemical transformations of coffee mucilage, generating key flavor precursors that shape the final cup profile (Bernardes, 2024; Hu et al., 2025). Among these, LAB play a particularly significant role through their metabolic activity, producing organic acids and volatile compounds that enhance acidity, sweetness, and overall flavor complexity (Duque-Buitrago et al., 2025).

Ecuador, with its high biodiversity and favorable climate, is emerging as a notable origin of specialty *C. arabica* production (Mihai et al., 2024), though it faces challenges from climate variability, plant diseases, and infrastructural limitations (Torres Castillo et al., 2020). The Intag Valley in northern Ecuador exemplifies eco-friendly agroforestry, where coffee is intercropped with banana, papaya, and cacao, supporting biodiversity while fostering diverse microbial communities on coffee cherries that influence fermentation outcomes (Venegas Sánchez et al., 2018; Todhanakasem et al., 2024).

Previously, our shotgun metagenomic study of three *C. arabica* varieties, Typica, Yellow Caturra, and Red Caturra, at two ripeness stages revealed a rich microbial diversity, including several LAB strains, with clear variety- and stage-specific community shifts (Tenea et al., 2025). While this study focused on the broader microbial diversity, the observed variations in the dominant bacterial genera and species across the coffee varieties strongly suggest that the LAB communities within these fermentations also differed, which would likely lead to variations in their respective metabolite profiles. For instance, *Levilactobacillus* and *Lactiplantibacillus* were dominant in Typica and Red Caturra, whereas *Acetobacter* was more abundant in Yellow Caturra (Tenea et al., 2025). These differences in LAB genera point toward potential variations in the types and quantities of metabolites produced. These findings suggest that varietal biochemical differences, particularly in sugars (sucrose, glucose, fructose), organic (citric, malic) acids, amino acids, and phenolic compounds, serve as distinct fermentation substrates that steer LAB metabolism (Mengesha et al., 2024). Moreover, the presence of anthocyanins in red and carotenoids in yellow varieties reflects divergent metabolic environments that further modulate LAB enzymatic activity and metabolite profiles (Tenea et al., 2025). Understanding these cultivar-specific microbial interactions is critical for optimizing fermentation to enhance desirable sensory traits (Silva et al., 2024).

LAB fermentation of coffee not only modulates its chemical composition, including chlorogenic acid, phenolics, and caffeine, but also enhances flavor, bioactivity, and functional properties, highlighting LAB potential to improve both the sensory quality and health benefits of specialty coffee (de Melo Pereira et al., 2016; Kim et al., 2024). Beyond flavor modulation, LAB also produce bioactive compounds such as peptides, organic acids, and phenolics with antioxidant properties, which can stabilize flavor precursors, protect aromatic molecules from oxidative degradation, and contribute to more complex and durable sensory profiles (de Carvalho Neto et al., 2018; Su et al., 2018; Tan et al., 2023). However, in this study, we comprehensively characterize the metabolomic profiles of six LAB strains isolated from *Coffea arabica* varieties, Typica, Yellow Caturra, and Red Caturra, harvested at both green and ripe stages. Capillary LC–MS/MS, coupled with a SWATH-based data-independent acquisition (DIA) strategy in positive electrospray ionization mode (ESI+), was employed to comprehensively characterize the metabolic outputs of LAB. This approach enabled the evaluation of strain- and cultivar-specific variations in metabolite production and their associations with flavor development, stress adaptation, and antimicrobial potential. In parallel, we employ ATR-FTIR spectroscopy to monitor structural and functional biochemical changes in the CFS, providing insights into the release and transformation of polysaccharides, proteins, lipids, and aromatic compounds during fermentation. Additionally, we assess total phenolic and flavonoid content in the CFS to explore their potential contributions to the antioxidant capacity, nutritional value, and sensory properties of fermented coffee. This integrated analytical approach allows for the identification of ripeness- and cultivar-dependent LAB metabolic signatures that enhance desirable sensory and functional traits. Ultimately, the outcome is a deeper understanding of how microbial fermentation modulates coffee biochemistry and flavor development, guiding the design of tailored, precision fermentation protocols. Our findings contribute to advancing microbial-based strategies for improving the sensory complexity and bioactive potential of Ecuadorian specialty coffees, with implications for enhancing their market value both locally and internationally.

## 2 Materials and methods

### 2.1 Bacterial isolates identification and culture

Six LAB isolates were obtained from fermented coffee cherries at two ripeness stages (green-red and green-yellow) from three *C. arabica* varieties: Typica (TYP), Yellow Caturra (CATY), and Red Caturra (CATR). For each sample, 500 g of cherries were collected from a coffee farm in the Intag Valley, Peñaherrera parish, Ecuador (0°21′0″ N, 78°44′0″ W), an area characterized by volcanic soil. Fruits were harvested in sterile bags according to ripeness stage, variety, and color (2 stages × 3 varieties × 2 colors), transported to the laboratory, and processed. The cherries were thoroughly washed and allowed to undergo natural fermentation in sterile flasks containing 100 mL of water at room temperature for 9 days. The ferment was inoculated onto MRS-agar containing 1% CaCO_3_ incubated at 37 °C for 48 h to select for LAB. Colonies were randomly picked from each fermented sample and purified by streaking on MRS-agar before being used for further studies. Using conventional 16S ARN sequencing these isolates were taxonomically identified. In brief, PCR amplification was performed using the primers 27F 5′ (AGA GTT TGA TCM TGG CTC AG) 3′ and 1492R 5′ (TAC GGY TAC CTTGTT ACG ACT T) 3′ (Weisburg et al., 1991), followed by a standard protocol with EF-Taq polymerase (SolGent, Korea). The amplicons were purified and sequenced using a PRISM BigDye Terminator v3.1 kit. Sequencing was carried out with primers 785F 5′ (GGA TTAGAT ACC CTG GTA) 3′ and 907R 5′ (CCG CAA TTC MTT TRA GTT T) 3′ (Muyzer et al., 1993), targeting the 16S RNA V3 region. The sequencing products were analyzed on an ABI Prism 3730XL DNA analyzer (Applied Biosystems, Foster City, CA, USA). Initial homology search was conducted using the megablast algorithm against the NCBI 16S database. Taxonomic classification was confirmed using the RDP Bayesian classifier algorithm (Wang et al., 2007) with 100 bootstrap replicates, integrated into the DADA2 package (Callahan et al., 2020) for high-resolution sample inference. Table 1 showed the identification codes, origin description of each sample and their NCBI assigned number. The strains were maintained as frozen stock cultures in MRS broth (Difco, Detroit, MI, USA).

**TABLE 1:** LAB strains isolated from fermented coffee cherries of different *Coffea arabica* varieties, including sample codes, species identification, strain codes, and corresponding NCBI accession numbers.

Group origin	Description	Code sample	Identification	Code strain	NCBI assigned number
*Coffea arabica* var. Typica	Fermented green cherries	B3	*Lactiplantibacillus plantarum*	TYPV 10	PV592383
	Fermented red cherries	B6	*L. plantarum*	TYPR 10	PV592384
*C. arabica* var. Yellow Caturra	Fermented green cherries	B9	*L. plantarum*	CRYV 8	PV592385
	Fermented yellow cherries	B10	*L. plantarum*	CRYA 2	PV592386
*C. arabica* var. Red Caturra	Fermented green cherries	B19	*Weissella confusa*	CATV 17	PV592387
	Fermented red cherries	B17	*L. pentosus*	CATR 10	PV592388

### 2.2 CFS extraction

To extract CFS, each LAB isolate was first cultured overnight in MRS broth at 37 °C for 24 h. Following incubation, the cultures were centrifuged at 13,000 × *g* for 30 min at 4 °C to separate the cell-free supernatant (CFS) from the bacterial cells. The resulting CFS was then passed through a 0.22 μm syringe filter (#STF020025H, Chemlab Group, Washington, DC, USA) and kept at 4 °C until further analysis. The CFS was lyophilized before use.

### 2.3 ATR-FTIR analysis

FTIR spectra of all freeze-dried samples were recorded using a Cary 630 FTIR Spectrometer (Agilent Technologies, Inc., USA) equipped with an ATR accessory and operated via Agilent MicroLab Software. Spectral acquisition was performed over the 4,000–650 cm^–1^ range, with 400 scans collected at a resolution of 4 cm^–1^ under ambient conditions. Freeze-dried MRS broth was used as the reference control.

### 2.4 Capillary LC-MS/MS procedure, SWATH data acquisition, processing and identification

Approximately 400 mg of each lyophilized CFS sample was rehydrated in 8 mL distilled water, refiltered through a 0.22 μm syringe filter, and centrifuged (15 min, 4 °C, 17,000 × *g*). The clear supernatant was transferred to vials for LC–MS/MS analysis on an AB SCIEX TripleTOF 5,600+ mass spectrometer coupled to a nanoACQUITY UPLC system equipped with a 5C18-CL-120 column and AB Sciex DuoSpray ion source. Chromatographic separation was achieved using a 90 min acetonitrile gradient (5%–80%, 0.1% formic acid) at 5 μL/min, with the column temperature maintained at 55 °C. The instrument was calibrated every three samples, maintaining mass accuracy within 4 ppm (calibration) and 20 ppm (up to 5 h). Electrospray ionization was performed in positive mode under the following parameters: GS1, 15; GS2, 0; CUR, 25; TEM, 0; ISVF, 5,500 V. Data were acquired in SWATH-MS mode using 60 variable windows (Xiao et al., 2021). MS1 scans covered 100–1,250 m/z (150 ms accumulation), while MS2 spectra were acquired in high-sensitivity mode from 100 to 2,000 m/z (30 ms accumulation), generating a 2 s duty cycle. Collision energy was optimized by Analyst TF 1.8.1 software with a 15 V spread. Untargeted metabolite identification was carried out using MS-DIAL v5.3.240719 and the MSP spectral kit database.^1^ MS-DIAL parameters included: retention time 1–90 min; MS1 range 100–1,250 Da; MS2 range 100–2,000 Da; minimum peak width, 5 scans; peak height threshold, 1,000 amplitude; smoothing level, 3 scans; MS2 spectrum cutoff, 10 amplitude; mass slice width, 0.05 Da; retention time tolerance, 0.1 min; MS1 tolerance, 0.01 Da; MS2 tolerance, 0.025 Da; matched spectrum ≥ 70%.

### 2.5 Prediction of enrichment pathways

The metabolites identified by LC–MS were linked to different enrichment pathways through Metabolomics Pathway Analysis version 6.0 (MSEA)^2^ (Pang et al., 2024). The enrichment tests utilize the globally recognized global test method to assess associations between metabolite sets and the outcome. This algorithm employs a generalized linear model to compute a “Q-stat” for each metabolite set, which is derived as the average of the Q values for each individual metabolite. The Q value represents the squared covariance between the metabolite and the outcome. The global test has demonstrated comparable or superior performance when benchmarked against various other widely used methods. The uploaded compounds list was converted by a built-in tool into common names, synonyms, and identifiers used in HMDB ID, PubChem, and KEGG databases. The Kyoto Encyclopedia of Genes and Genomes (KEGG) database^3^ was utilized to map these metabolites and determine their roles in metabolic pathways (Kanehisa et al., 2024).

### 2.6 Quantification of phenolic secondary metabolites

The supernatant was subjected to a liquid-liquid extraction in ethyl acetate (1:1, v: v) and evaporated to dryness. The residue was taken up in 70% ethanol in a volume like the sample volume used for the liquid-liquid extraction.

#### 2.6.1 The total phenolic content (TPC) assay

Following the procedure outlined by Corbu et al. (2022), the Folin-Ciocalteu assay was used to quantify the total phenolic content (TPC). Folin-Ciocalteu reagent (0.1 mL), distilled H_2_O (1.8 mL), and saturated Na_2_CO_3_ (0.1 mL) were combined with an aliquot. To develop the color, the tubes were vortexed for 15 s and then left in the dark for 60 min. At 765 nm, the absorbance was then measured. The same circumstances as the samples were used to create a standard curve with varying gallic acid concentrations (R^2^ = 0.9972). The amount of TPC was measured in milligrams of gallic acid equivalent per milliliter of extract (mg GAE/L). Three separate analyses were conducted.

#### 2.6.2 The total flavonoid content (TFC) assay

Using the AlCl_3_ method as described by Corbu et al. (2022), the TFC assay was assessed. To put it briefly, 0.12 mL of 2.5% AlCl_3_ and 0.1 mL of 10% sodium acetate were combined with 0.1 mL of the sample/standard solution, and the final volume was adjusted to 1 mL using 50% ethanol. After that, the samples were vortexed and left for 45 min in the dark. At λ = 430 nm, absorbances were measured. Various quercetin concentrations were used to create a standard curve (R^2^ = 0.9996). The total flavonoid concentration was reported as mg of quercetin equivalent per L of extract (mg QE/L). Three separate analyses were conducted.

### 2.7 Statistical analysis

Based on measurements made in triplicate, all results were displayed as mean ± standard deviation (SD). GraphPad Prism version 10 (GraphPad Software, San Diego, CA, USA) was used for statistical analyses. The effects of metabolic extracts and culture media (MRS) were evaluated using a pooled variance technique, with Tukey’s *post-hoc* test for multiple comparisons and a one-way analysis of variance (ANOVA) for differences in polyphenols and flavonoids content. The assumptions of ANOVA were verified by assessing normality with the Shapiro–Wilk test and homogeneity of variances with the Brown–Forsythe test. The threshold for statistical significance was *p* < 0.05 according to GraphPad Prism software.

## 3 Results and discussion

### 3.1 LAB strain-specific metabolic profile shape coffee fermentation flavors

**FIGURE 1:**
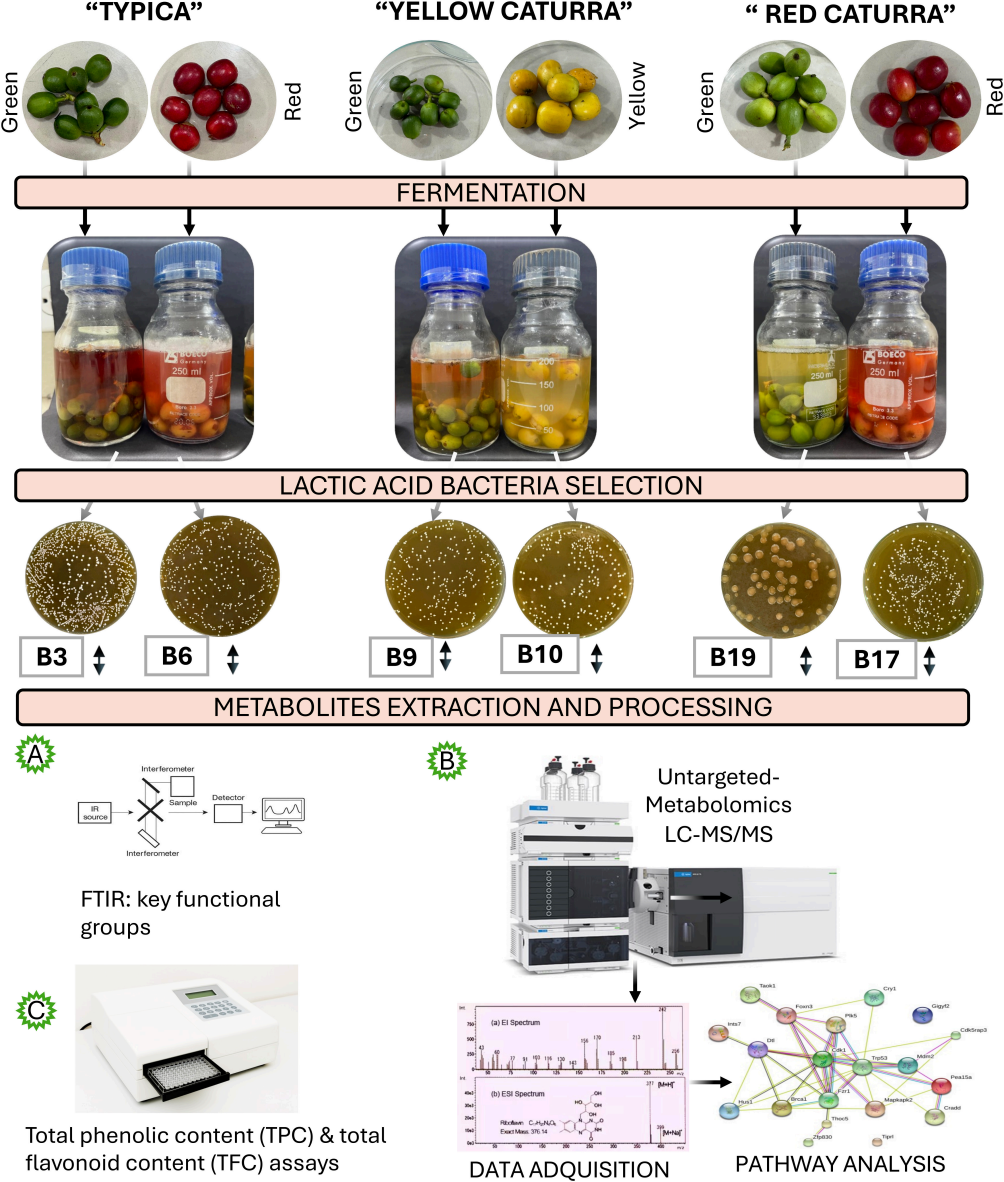
Workflow for metabolomic profiling of LAB isolated from Coffea arabica cultivars. Fermented isolates were screened for bioactive potential using ATR-FTIR **(A)**, untargeted LC-MS/MS (ESI^+^) **(B)**, and colorimetric assays **(C)**.

To elucidate the contribution of LAB to coffee fermentation, we applied an integrative metabolomic workflow combining untargeted LC–MS/MS (ESI+), ATR-FTIR spectroscopy, and colorimetric assays. Six representative isolates (B3, B6, B9, B10, B17, and B19) derived from Typica, Yellow Caturra, and Red Caturra cultivars at different ripening stages were selected for comparative analysis (Figure 1).

**FIGURE 2:**
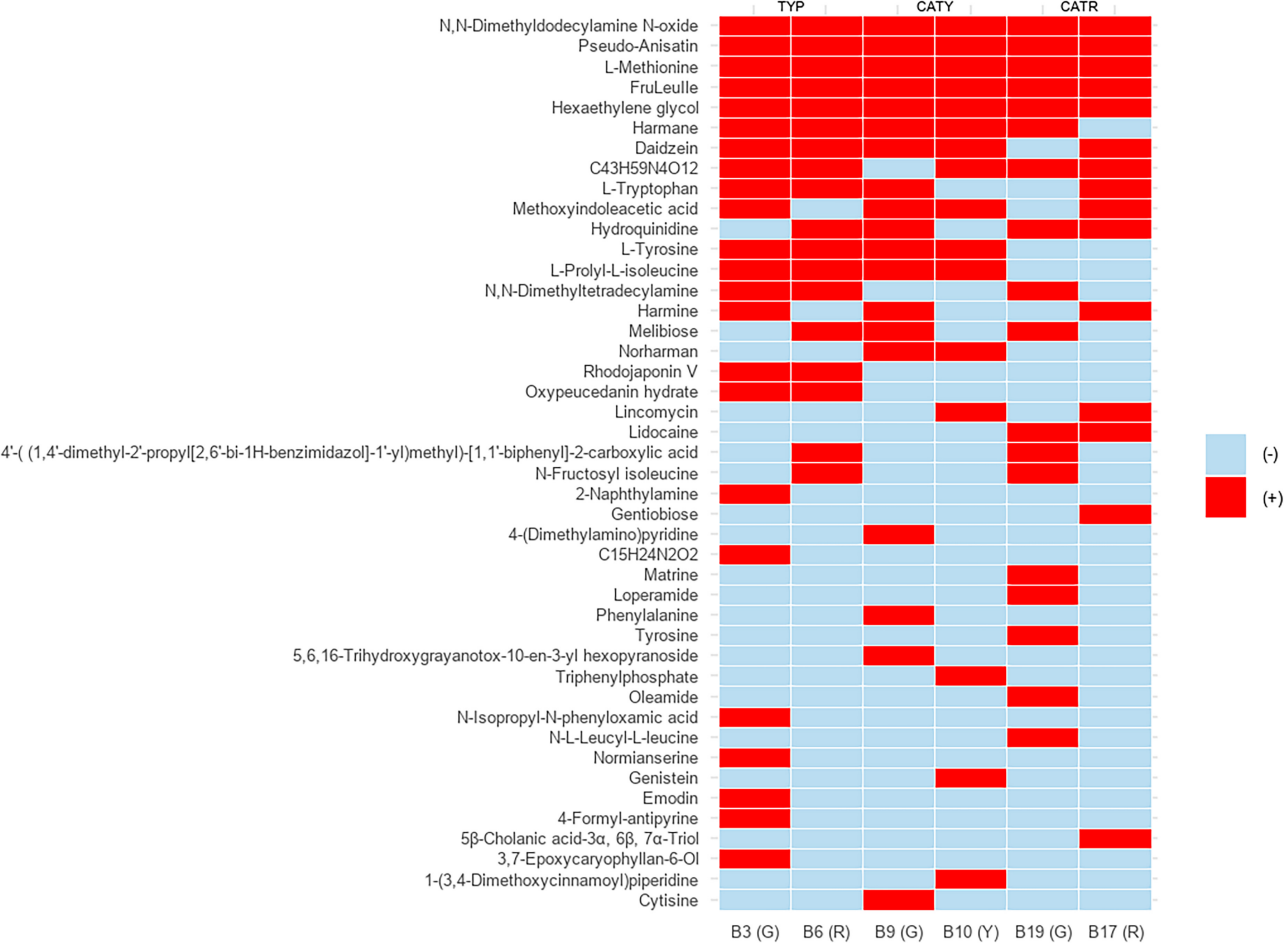
Presence–absence heatmap of detected metabolites across LAB strains isolated from different coffee cherries varieties (CATR, CATY, TYP). Red bars indicate the presence (+) of specific compounds, while absence is denoted by blue cells (–).

Untargeted LC–MS/MS profiling, visualized as a binary presence–absence heatmap (Figure 2), revealed highly strain-specific metabolite distributions, reflecting distinct biosynthetic and catabolic capacities. Key metabolite classes included aromatic and sulfur-containing amino acids (L-tyrosine, L-tryptophan, L-methionine), disaccharides (melibiose, gentiobiose), β-carboline alkaloids (harmane, norharmane, harmine), isoflavones (daidzein, genistein), and low-molecular-weight peptides such as FruLeuIle. These compounds are well documented for their roles in modulating umami, bitterness, aromatic complexity, and overall sensory perception in fermented foods (Parthasarathy et al., 2018; Haile and Kang, 2019; Schieber and Wüst, 2020).

Besides, metabolite origin may derive from both *de novo* biosynthesis and enzymatic biotransformation of medium-derived substrates. LAB are equipped with proteases, glycosidases, and esterases that hydrolyze proteins, polysaccharides, and phenolic esters into bioactive units (Virdis et al., 2021). Thus, the detected pool of peptides, disaccharides, and aromatic amino acids likely reflects a dual contribution of biosynthetic activity and substrate remodeling. This metabolic plasticity underscores the capacity of LAB to enrich coffee fermentation with structurally diverse, flavor-active metabolites under nutrient-rich conditions.

Drawing from these strain-specific metabolic signatures, aromatic amino acids emerged as pivotal contributors to flavor formation. L-tyrosine derivatives such as *p*-cresol and tyramine impart subtle spicy and woody nuances (Li et al., 2020), while indole compounds derived from L-tryptophan enhance floral and fruity notes (Xiao et al., 2023). L-methionine, consistently detected across isolates, provides sulfur precursors for volatile compounds that add savory and complex dimensions, like those found in dairy and cured meat products (Bonnarme et al., 2000). Together, these sensory-active metabolites highlight how LAB adapt to the phytochemical richness of *C. arabica* cherries, translating varietal and environmental differences in sugars, amino acids, and polyphenols into distinctive coffee flavor profiles (Todhanakasem et al., 2024).

In addition to amino acid–derived volatiles, β-carboline alkaloids were identified (Supplementary Figure 1A). Harmane, typically associated with roasted or nutty notes from Maillard reactions (Piechowska et al., 2019), was detected in CFS of LAB representing the first report of harmala-like alkaloids in this group. Traditionally linked to *Peganum harmala* (Moloudizargari et al., 2013), their occurrence here suggests that LAB may convert tryptophan-derived intermediates into β-carbolines via tryptophan decarboxylase and monoamine oxidase activities (Zuo et al., 2025). This enzymatic route involves tryptamine intermediates and oxidative steps leading to β-carbolines through Pictet–Spengler condensation (Matsui et al., 2018; Zhao et al., 2020). The enrichment of tryptophan and caffeine in coffee berries may provide precursors or cofactors for such pathways, supporting niche-driven metabolic adaptation in LAB. This aligns with previous reports of genomic plasticity in *L. plantarum* within plant-associated environments (Siezen et al., 2010; Martino et al., 2016). While microbial β-carboline biosynthesis remains rare, it has been demonstrated in engineered *E. coli* and *Streptomyces* expressing Pictet–Spenglerases (Kishimoto et al., 2016), highlighting the novelty of our observation of spontaneous β-carboline production in LAB.

Isoflavones were also detected, with daidzein consistently present in *Lactiplantibacillus* strains (B3, B6, B9, B10, B17) and genistein uniquely in B10 (Supplementary Figures 1B, C). These compounds, known for antioxidant activity and astringency, point to roles beyond flavor modulation (Kim, 2021). Notably, B17 (*L. pentosus*) showed enriched disaccharide (gentiobiose) and alkaloid (harmine) profiles, indicating enhanced capacity to modulate sweetness, bitterness, and antimicrobial potential (Zhao et al., 2020; Kishimoto et al., 2016).

Moreover, all isolates produced hexaethylene glycol (Supplementary Figure 1D), a surfactant-like compound that, while not flavor-active, may influence aroma solubility and release kinetics (Wijaya et al., 2016). Among Typica-derived strains, B3 and B6 exhibited the broadest metabolite repertoires, underscoring their potential as starter cultures for improving mouthfeel and sensory complexity. In contrast, B9 (*L. plantarum*) and B19 (*W. confusa*) showed narrower but distinct metabolic signatures, suggesting application in niche fermentations with unique sensory outputs (Gao et al., 2021).

Finally, metabolites detected in *Weissella* B19 further underscore functional versatility. 5β-Cholanic acid-3α,6β,7α-triol, a bile acid derivative, may confer antimicrobial activity and bile resistance (Begley et al., 2006), while gentiobiose supports prebiotic effects through selective stimulation of beneficial LAB and short-chain fatty acid production (Mussatto and Mancilha, 2007; Ucar et al., 2020). These features highlight the dual role of *W. confusa* in probiotic resilience and host–microbe interactions.

Taken together, these findings demonstrate that LAB isolated from coffee cherries possess a diverse metabolic repertoire capable of generating flavor-active, antioxidant, antimicrobial, and prebiotic compounds. Such results not only support the application of indigenous LAB strains for precision fermentation but also emphasize the influence of phytochemical-rich coffee environments in shaping microbial secondary metabolism.

### 3.2 Peptides production: a microbial adaptation to local substrate

Coffee plants grown at higher altitudes are known to accumulate greater concentrations of sugars and polyphenols, contributing to enhanced flavor complexity and unique chemical profiles in the fruit (Tolessa et al., 2017). Red and yellow cherries, which represent more advanced stages of maturation, are characterized by higher concentrations of readily fermentable sugars and free amino acids (Hall et al., 2022). Based on this study, all strains produce small peptides in the CFS, may have undergone selective metabolic adaptation to exploit these nutrient-rich environments. The consistent detection of the tripeptide FruLeuIle suggests a potential link between coffee cherry and microbial peptide biosynthesis during fermentation (Supplementary Figure 1E). This observation indicates a metabolically adaptive response of LAB to the distinct phytochemical environments associated to cherry ripeness.

*Lactobacillus* species are well known to secrete a variety of extracellular and intracellular peptidases, which hydrolyze host proteins into small bioactive peptides (Pessione and Cirrincione, 2016). During the natural ripening of coffee cherries, proteolytic processes are known to intensify, leading to the accumulation of free amino acids and small peptides because of both plant enzymatic activity and microbial contributions (Bastian et al., 2021). Besides, the production of specific bioactive peptides may play a role in microbial signaling (quorum sensing), stress resistance, and the formation of precursor molecules for volatile flavor compounds, thereby contributing to the sensory attributes of fermented coffee (Bastian et al., 2021). Moreover, previous studies have demonstrated that peptide accumulation in LAB can be influenced by the availability of carbohydrates and the presence of phenolic compounds two factors that vary with fruit maturity (Zhao et al., 2021; Padonou et al., 2022). In this context, the presence of the small peptide FruLeuIle in strains associated with mature cherries could be interpreted as a biosignature of substrate-driven metabolic specialization. This further underscores the role of plant–microbe coevolution and ecological selection in shaping the metabolic repertoire of LAB, with direct implications for the enhancement of coffee flavor and quality through fermentation. This supports the hypothesis that plant-microbe cohabitation in niche environments fosters functional diversification in microbial metabolism (Junkins et al., 2022).

Moreover, the detection of lincomycin in the CFS of B10 and B17 from Caturra variety yellow and red mature cherries, suggests a possible case of metabolically induced antibiotic production or the synthesis of structurally related analogs. While lincomycin is traditionally produced by *Streptomyces* spp., recent studies have highlighted the metabolic plasticity of LAB in response to complex plant-derived substrates, particularly under selective ecological pressures (Molina et al., 2025). The nutrient-rich composition of mature coffee cherries characterized by high concentrations of fermentable sugars, free amino acids, and polyphenolic compounds (Hall et al., 2022), may serve as biochemical cues that activate cryptic or horizontally acquired biosynthetic pathways. Such pathways could facilitate the production of antimicrobial compounds, conferring a competitive advantage by modulating microbial community dynamics during fermentation (Bastian et al., 2021). Nonetheless, the consistent detection of such metabolites in CFS derived from mature cherry fermentations underscores the potential for substrate-induced metabolic specialization in LAB, with implications for both microbial ecology and the development of bioactive compounds in coffee (Zhao et al., 2021; Padonou et al., 2022).

### 3.3 Vibrational signatures of LAB-derived metabolites as a perspective on the modulation of aroma and functionality through FTIR spectroscopy

**FIGURE 3:**
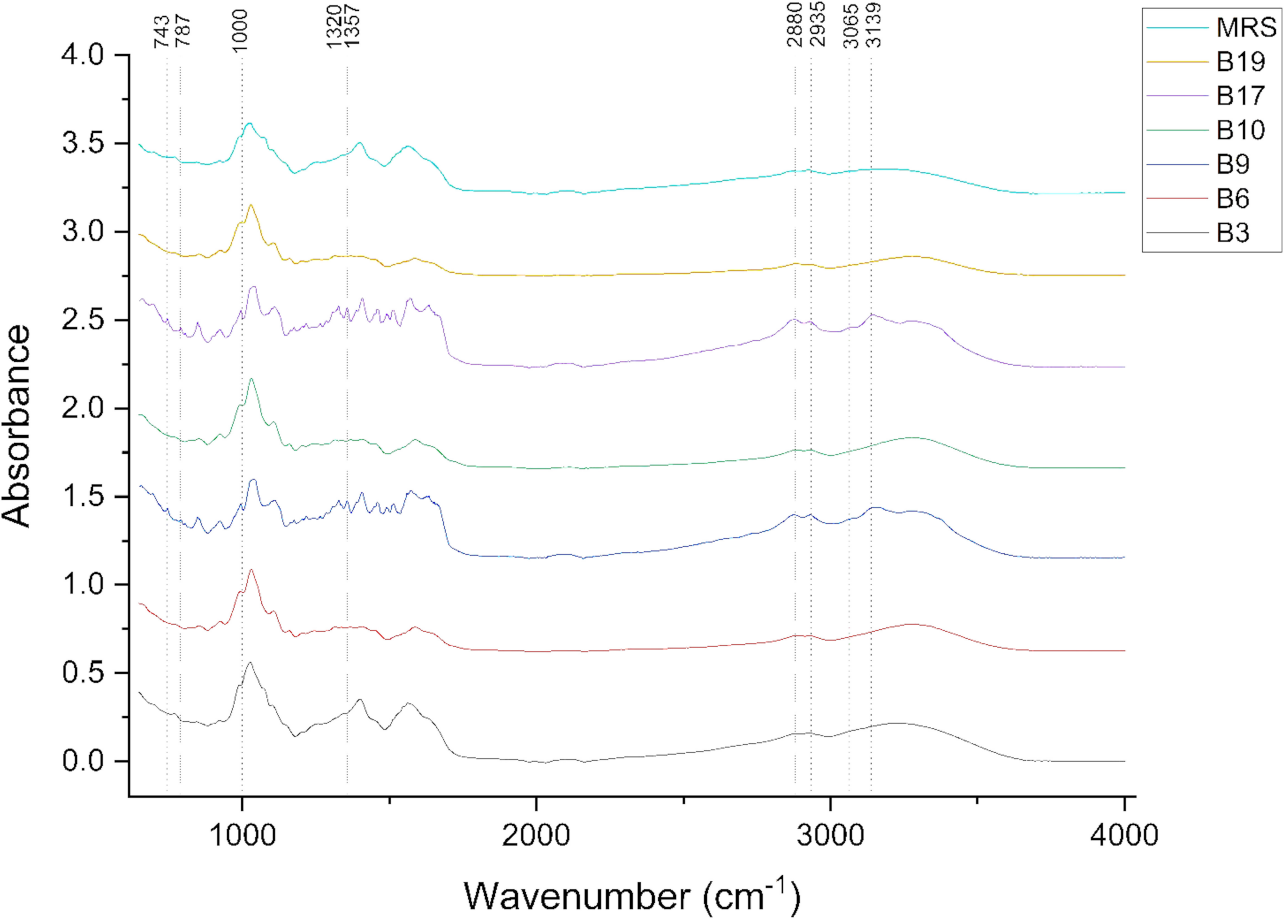
FTIR spectral profiles of LAB-derived extracellular metabolites compared to uninoculated MRS medium.

The FTIR spectra of the extracellular metabolites produced by the LAB strains (Figure 3) were significantly different from the un-inoculated and lyophilized MRS broth in certain spectral regions, demonstrating biochemical changes caused by fermentation.

In the region 690–1,000 cm^–1^ (fingerprint for carbohydrates), the bands at 698–808 cm^–1^ in the free cell supernatant of the samples, which were absent in the MRS medium spectrum, suggest the presence of new compounds, possibly polysaccharides or proteins. The strong bands in the region of 929–1,000 cm^–1^, common to all samples, correspond to the C-O-C and C-O vibrations characteristic of polysaccharides (β-glycosidic bond) or phosphates, suggesting that the microbial cells did not fully metabolize the carbohydrate components present in the broth (Chen et al., 2025; Gieroba et al., 2023; Jastrzebski et al., 2011).

In the region between 1,000 and 1,372 cm^–1^, polysaccharides were confirmed by C-O stretching vibrations in the range of 1,000–1,100 cm^–1^, while CO deformation in condensed COC structures was highlighted in the range of 1,112–1,164 cm^–1^, which are specific to carbohydrates and/or peptides (Xu et al., 2013; Khadivi et al., 2022). Only for samples B6, B10, and B19 was a band at 1,164 cm^–1^, suggesting the presence of distinct extracellular polysaccharides with a more complex structure (polysaccharides with a higher degree of branching or specific types of glycosidic linkages), which differentiates them from the other samples (Liu et al., 2021). For samples B9 and B17, a specific band at wavenumbers 1,180 cm^–1^ and 1,265 cm^–1^ was identified, which can be attributed to phenolic C-O stretching (Agatonovic-Kustrin et al., 2020). In the case of B17 sample, a band at 1,289 cm^–1^ was identified and attributed to the C-O stretching vibration of the amide group and the C–N stretching vibration in the pyrrolidine ring (Yang et al., 1997; Fu et al., 1994; Li et al., 2013; Jeevaratnam et al., 2015). The changes in the region 1,343–1,372 cm^–1^, where additional bands are detected around 1,357 cm^–1^ for samples B9 and B17, are attributed to the symmetric O-C-O vibration (Chico-Mesa et al., 2025).

The amide (Amide I and II) and aromatic regions are between 1,400 and 1,700 cm^–1^. The bands in the range of 1,470–1,570 cm^–1^ indicate the presence of carboxyl and amide groups (Chatterley et al., 2022; Ji et al., 2020). The deformation vibrations of the CH_2_ or CH_3_ group, which are unique to lipids, but found also in proteins, were responsible for the absorption maxima that were seen at wavenumbers 1,402 and 1,451 cm^–1^ in the uninoculated media. The O–H bending vibration of carboxylic acids can be shown by the peak at 1,402 cm^–1^ (Yang et al., 2025). Additionally, the intense band around the value of 1,562–1,579 cm^–1^ suggests the presence of amide-type vibrations (Amide II), characteristic of proteins or extracellular peptide compounds (Ghafoor and Butt, 2025). The presence of the band at 1,592 cm^–1^, characteristic of C = C vibrations in aromatic rings for samples B6, B10, and B19, indicates the presence of aromatic amino acids such as phenylalanine, tyrosine, or tryptophan, or phenolic compounds (Amber et al., 2023; Tomasetig et al., 2024). The bands in the range of 1,600–1,700 cm^–1^ are associated with C = O vibrations of the amide type (Amide I), highlighting the presence of extracellular proteins or peptides (Sadat and Joye, 2020). Only for sample B9 was a specific band identified at a wavenumber of 1,655 cm^–1^, associated with an extracellular protein structure with a possible α-helix type structure (Barth, 2007; Sizeland et al., 2018).

Bands in the range of 2,923–2,933 cm^–1^, attributed to C-H stretching vibrations, suggest the existence of lipid and carbohydrate structures (Portaccio et al., 2023). A broad absorption around the values of 3,200–3,300 cm^–1^ (amide A) was present in both free cell supernatant samples and the uninoculated medium, but slight shifts in the peaks indicate a change in the content of extracellular polysaccharides or glycoproteins (Synytsya and Novak, 2014; Das et al., 2021; Tatulian, 2019). FTIR spectrum analysis confirms that the lactic acid bacteria strains secreted extracellular metabolites into the culture medium, particularly polysaccharides and proteins, thereby altering the initial composition of the MRS medium.

The evaluation of FTIR spectra showed that, during fermentation, LAB strains actively release extracellular polysaccharides, peptides, proteins, and phenolic and aromatic compounds. These biochemical changes distinguish the fermented matrix from the original MRS environment and demonstrate the distinct metabolic adaptations of the bacteria. Samples B6, B10, B17, and B19 showed unique markers in the individual spectrum with different aromatic compounds or structural polysaccharides, highlighting their ability to control functional bioactivity, texture, and aroma release under coffee fermentation conditions.

### 3.4 Metabolic pathway enrichment profiles of coffee-origin LAB strains across Typica, Caturra Yellow, and Caturra Red varieties

#### 3.4.1 Metabolic enrichment profiles of Typica-derived strains (B3 and B6)

**FIGURE 4:**
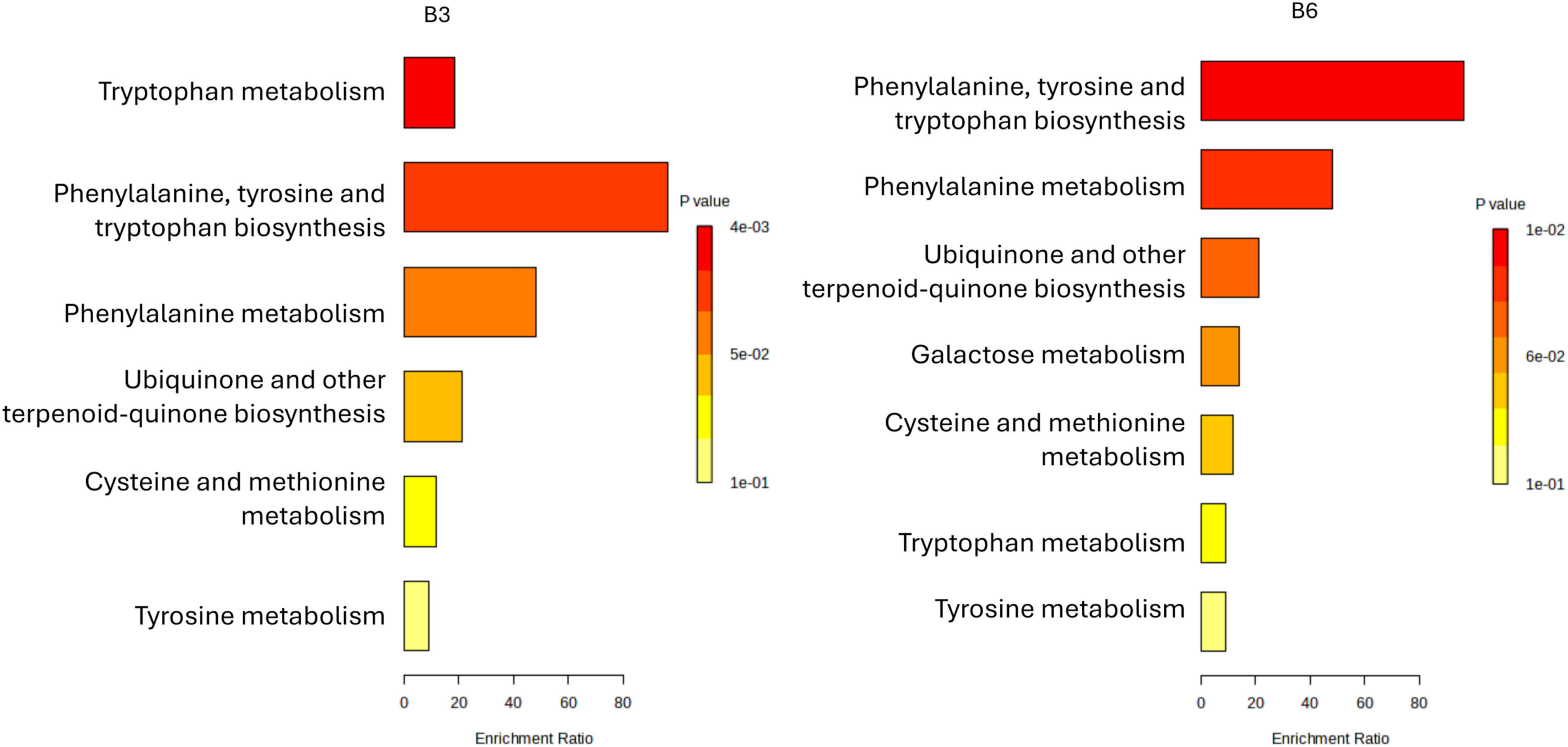
Metabolite set enrichment overview associated with LAB strains isolated from *C. arabica* var. Typica (B3 and B6). Enrichment bar plots illustrate the significantly overrepresented chemical classes among metabolites produced by each strain. The *x*-axis denotes the enrichment ratio, reflecting the degree of overrepresentation relative to background levels, while bar coloration corresponds to statistical significance (*p*-value), with red indicating the highest significance and yellow the lowest.

The metabolic pathway enrichment analysis of samples B3 and B6 revealed pronounced activation of aromatic amino acid and carbohydrate metabolism pathways (Figure 4). Both strains showed elevated phenylalanine and tryptophan biosynthesis and metabolism, precursors of phenols, indoles, and other flavor-active compounds (Evangelista et al., 2014), suggesting their potential to enhance sensory profiles during coffee pulp fermentation. Enrichment in novobiocin biosynthesis and tyrosine metabolism further indicated active secondary metabolism, possibly contributing to bioactivity and functional properties of the fermented matrix (Supplementary Figure 2). Notably, B6 uniquely exhibited significant enrichment in galactose metabolism, pointing to a superior capacity for carbohydrate catabolism that could modulate fermentation kinetics and direct metabolic flux toward flavor precursors (Li et al., 2021). Both strains also showed enrichment in one-carbon metabolism via folate and sulfur amino acid pathways, supporting roles in methylation reactions and sulfur-containing volatile production, key to flavor complexity and oxidative stability (Liu et al., 2008). Together, these results suggest that B3 and B6 drive flavor development through aromatic amino acid and sulfur metabolism, with B6 emerging as a particularly promising candidate due to its expanded sugar metabolism and potential to improve both flavor quality and functional value in coffee by-products (Hall et al., 2022; Todhanakasem et al., 2024).

#### 3.4.2 Metabolic enrichment profiles of Caturra Yellow-derived strains (B9 and B10)

**FIGURE 5:**
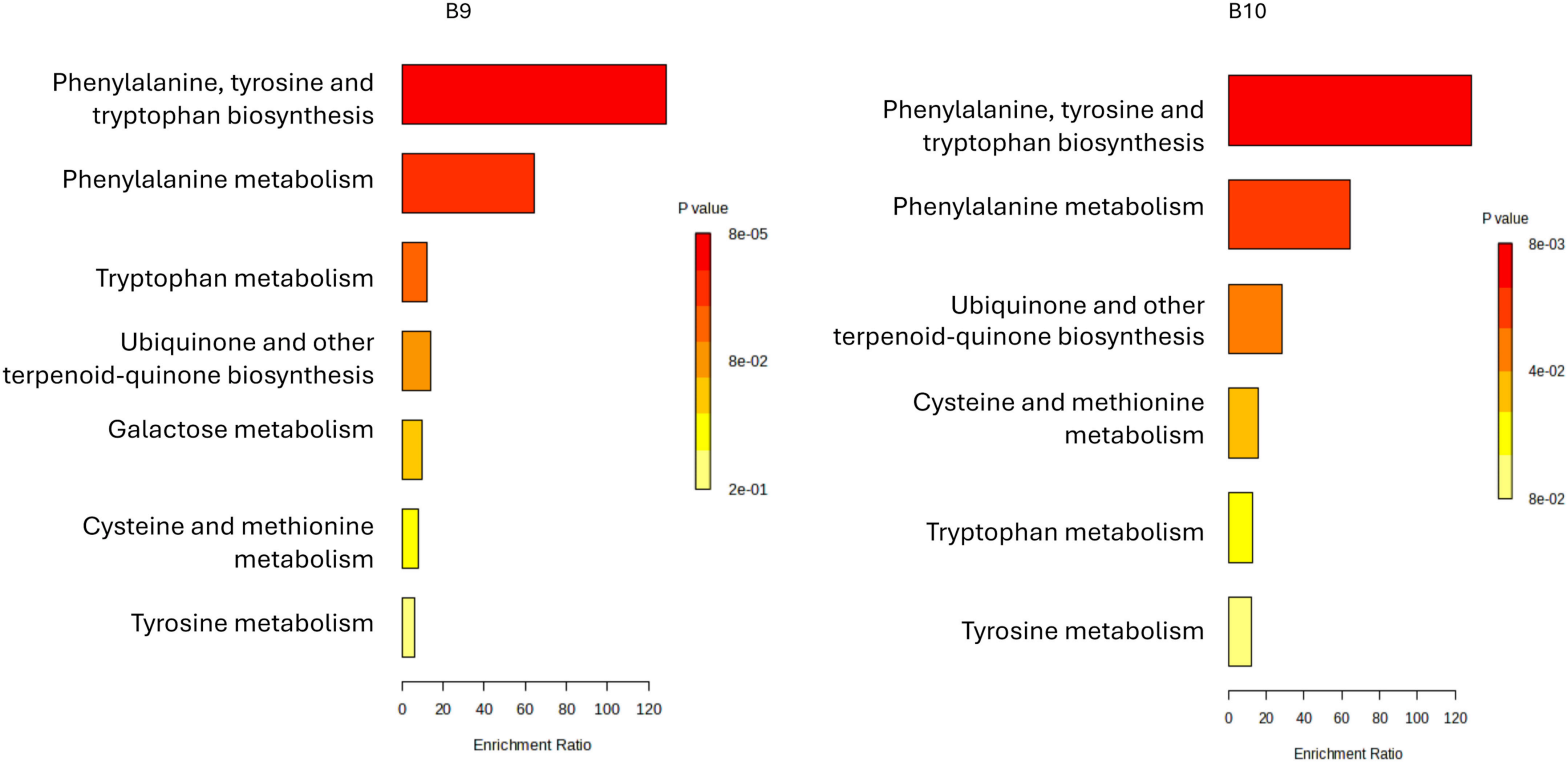
Metabolite set enrichment overview associated with LAB strains isolated from *C. arabica* var. Caturra Yellow (B9 and B10). Enrichment bar plots illustrate the significantly overrepresented chemical classes among metabolites produced by each strain. The *x*-axis denotes the enrichment ratio, reflecting the degree of overrepresentation relative to background levels, while bar coloration corresponds to statistical significance (*p*-value), with red indicating the highest significance and yellow the lowest.

The metabolite set enrichment analysis of B9 and B10 revealed distinct but overlapping metabolic reprogramming (Figure 5). Both strains showed strong enrichment in phenylalanine, tyrosine, and tryptophan biosynthesis and phenylalanine metabolism, highlighting upregulation of aromatic amino acid pathways that provide precursors for alkaloids and phenolic compounds linked to stress adaptation and signaling (Dodd et al., 2017). B9 exhibited particularly strong enrichment in tryptophan metabolism (Supplementary Figure 3), suggesting elevated production of indole-derived compounds with roles in plant defense and growth regulation (Tennoune et al., 2022). Both strains also showed enrichment in ubiquinone and terpenoid-quinone biosynthesis, as well as moderate activation of cysteine, methionine, and galactose metabolism, pointing to adjustments in redox homeostasis and energy metabolism (Paul et al., 2018). While B9 displayed stronger activation of aromatic amino acid and tryptophan pathways, B10 (*L. plantarum*) exhibited a broader but less intense enrichment profile, potentially reflecting different stress responses and niche adaptations. Pathway mapping for B10 was limited by the smaller number of annotated metabolites. Overall, these findings underscore strain-specific metabolic strategies of coffee-associated LAB, shaped by adaptation to the biochemical challenges of coffee fermentation.

#### 3.4.3 Metabolic enrichment profiles of Caturra Red-derived strains (B17 and B19)

**FIGURE 6:**
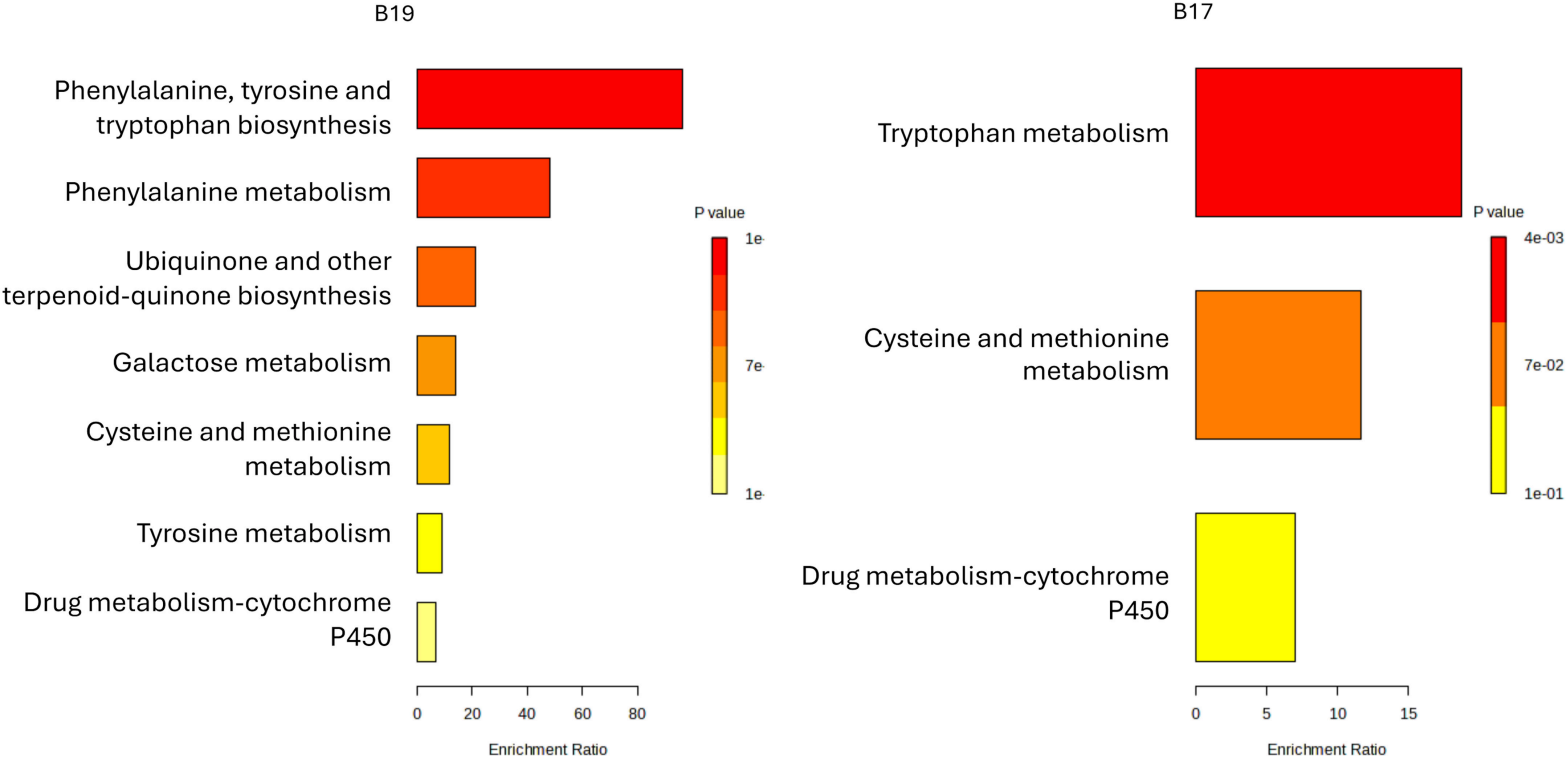
Metabolite set enrichment overview associated with LAB strains isolated from *C. arabica var*. Caturra Red (B17 and B19). Enrichment bar plots illustrate the significantly overrepresented chemical classes among metabolites produced by each strain. The *x*-axis denotes the enrichment ratio, reflecting the degree of overrepresentation relative to background levels, while bar coloration corresponds to statistical significance (*p*-value), with red indicating the highest significance and yellow the lowest.

The metabolite enrichment profiles of B17 (*L. pentosus*) and B19 (*W. confusa*), reveal distinct metabolic strategies that align with their ecological roles and potential functional applications in coffee fermentation (Figure 6). B17 demonstrates significant enrichment in pathways associated with phenylalanine, tyrosine, and tryptophan biosynthesis, as well as phenylalanine metabolism, ubiquinone and other terpenoid-quinone biosynthesis, and galactose metabolism. This broad enrichment suggests that B17 possesses an active aromatic amino acid metabolism capable of generating precursor compounds for flavor-active phenolics and volatiles (Maeda and Dudareva, 2012). The engagement of galactose metabolism is particularly relevant, considering that coffee mucilage contains substantial amounts of galactose-rich polysaccharides (de Soares da Silva et al., 2023), indicating B17 adaptability in utilizing coffee-derived sugars during fermentation. Moreover, the activation of ubiquinone and terpenoid-quinone biosynthesis in B17 implies a heightened electron transport and redox balancing capacity, which could improve resilience under oxygen-variable conditions typical of spontaneous coffee fermentations (De Bruyn et al., 2016). This redox activity may also indirectly influence the fermentation’s microbial ecology and oxidative processes involved in flavor development. Nonetheless, similar with the sample B10, the limited number of compounds mapped to known metabolic pathways precluded the generation of a pathway profile for B17. In contrast, B19 exhibits a more focused metabolic enrichment, with dominant activation of tryptophan metabolism, cysteine and methionine metabolism, and drug metabolism (Figure 6 and Supplementary Figure 4). The sulfur amino acid metabolism reflects an adaptive stress response mechanism, enabling *Lactobacillus* sp. to manage oxidative stress and thrive in the challenging conditions of fermentation (Papadimitriou et al., 2016). Additionally, cytochrome P450-related activity in B19 points to its role in detoxification and secondary metabolism, which can support fermentation stability by neutralizing inhibitory compounds (Wszołek, 2022). From a functional perspective, these metabolic signatures suggest B17 (*L. pentosus*) is well-suited for early-stage fermentation, where its carbohydrate catabolism, redox modulation, and aromatic amino acid metabolism can actively shape flavor and microbial succession. This aligns with previous reports highlighting *Weissella* spp. as early colonizers in coffee and cacao fermentations, contributing to initial sugar breakdown and acid production (De Bruyn et al., 2016). In contrast, B19 (*W. confusa*) appears more adapted to late-stage fermentation, where its stress tolerance and detoxification systems maintain process stability and microbial homeostasis. *L. plantarum* is known for its robustness and versatile metabolism in diverse fermentation systems, including vegetable and cereal fermentations (Filannino et al., 2018). Considering the coffee origin of both strains, leveraging their complementary metabolic traits could enhance the quality and consistency of coffee fermentation. Specifically, B17 ability to metabolize galactose and activate aromatic pathways positions it as a driver of early biochemical transformations that define coffee sensory profile, while B19 could ensure microbial robustness and redox balance in later stages. Therefore, a sequential or co-culture application of B17 and B19 offers a promising strategy for optimizing coffee fermentation, improving both flavor complexity and process reliability.

### 3.5 Strain-specific metabolite enrichment revealed by pathway analysis

**FIGURE 7:**
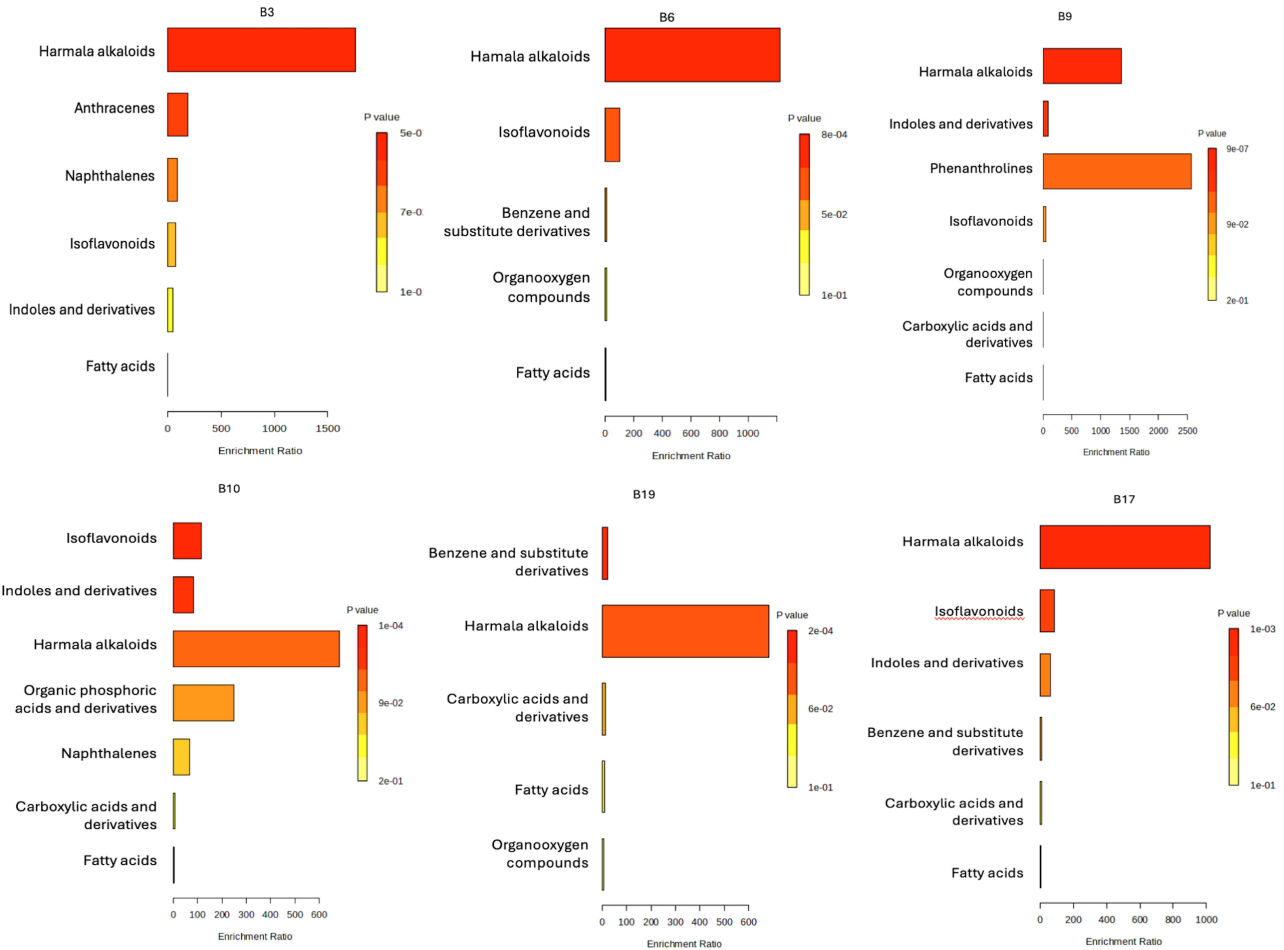
Enriched metabolic pathways based on KEGG analysis detected in LAB isolates. Enriched classes include harmala alkaloids, indoles and derivatives, isoflavonoids, organooxygen compounds, and fatty acids, with strain-specific differences in enrichment ratios and significance levels (*p*-values). These results highlight the metabolic diversity and functional specialization of LAB strains in coffee fermentation.

The metabolomic profiles of the CFS from six bacterial strains (B3, B6, B9, B10, B17, and B19) revealed distinct enrichment patterns based on metabolite set analysis (Figure 7). Notably, the strains could be divided into two main species-specific metabolic profiles:

#### 3.5.1 *Lactiplantibacillus* strains enrich harmala alkaloids, isoflavonoids, and indole derivatives

CFS extracted from B3, B6, B9, B10, and B17 exhibited significant enrichment in harmala alkaloids, isoflavonoids, and indole derivatives (Figure 7). LAB associated with *Coffea arabica* may either produce these compounds as part of their secondary metabolism or possess specific metabolic pathways to tolerate or transform alkaloid compounds. Similar observations have been reported in microbial communities inhabiting alkaloid-rich environments, such as cocoa fermentations, where the presence of alkaloids acts as a selective pressure, shaping microbial community structure and function (Picon et al., 2024). This finding suggests a potential metabolic adaptation of the LAB strains to the chemical environment of coffee cherries, known to be rich in alkaloids and phenolic compounds. Among them, B9 showed a distinctive signature in aromatic amino acid metabolism (phenylalanine, tyrosine, tryptophan) but still followed the general trend of *Lactobacillus* strains producing secondary metabolites. The strong enrichment of β-carboline derivatives (harmala alkaloids) suggests an active metabolism of tryptophan. *Lactobacillus* species are known to metabolize tryptophan into bioactive compounds such as indole-3-lactic acid, indole-3-acetic acid, and potentially β-carbolines via microbial secondary metabolism (Hou et al., 2023). This ability could enhance the functional properties of fermented coffee by producing neuroactive and antioxidant metabolites. Moreover, the detection of isoflavonoid derivatives is consistent with *Lactobacillus* strains ability to hydrolyze glycosides into aglycones via β-glucosidase activities (Strahsburger et al., 2017). Such bioconversions not only improve the bioavailability of polyphenols but also contribute to the sensory characteristics of the fermented product. The metabolic signatures of B9 emphasized the metabolism of phenylalanine, tyrosine, and tryptophan, pointing toward a highly active aromatic amino acid biosynthesis and catabolism pathway. The secondary metabolites enriched by *Lactobacillus* strains, particularly indole derivatives and isoflavonoids, are likely to contribute to the development of fruity, floral, and sweet aromatic profiles in fermented coffee. In addition, β-carbolines have been reported to impart subtle bitterness and complexity, potentially enhancing mouthfeel and the perceived body of the coffee beverage.

#### 3.5.2 Distinct metabolic signature in *Weissella* strain

The *W. confusa* strain CATV17 (sample B19) exhibited a different metabolic enrichment pattern (Figure 7). The enrichment pathway was focused on benzene derivatives and carboxylic acids rather than secondary metabolites like harmala alkaloids or indoles. *Weissella* species are known for their proteolytic activities and the ability to metabolize simple aromatic structures derived from amino acids or plant phenolics (Fusco et al., 2015). The limited presence of harmala alkaloids and lower enrichment ratios for complex secondary metabolites suggest a metabolism more oriented toward basic energy acquisition and simple aromatic transformations, rather than elaborate secondary metabolism. The metabolic profile of B19 suggests a potential contribution to acidity and fresh, clean flavors in fermented coffee, dominated by organic acid production and simple aromatic transformations. However, the lack of enriched complex secondary metabolites indicates that *Weissella* alone might not provide the same degree of aromatic complexity (e.g., floral, fruity notes) compared to *Lactiplantibacillus* strains.

#### 3.5.3 Minor but conserved pathways across strains

All strains exhibited low yet measurable enrichment in pathways related to fatty acyl metabolism, organooxygen compounds, and naphthalene degradation, likely reflecting partial catabolism of plant-derived aromatics from the coffee pulp (Figure 7). The strain-specific metabolomic profiles observed here are consistent with previous reports showing that microbial secondary metabolism is highly strain-dependent and shaped by environmental factors, particularly the phytochemical composition of the host plant (Lippolis et al., 2023). Notably, the enrichment of isoflavonoids across strains suggests a conserved metabolic response to phenolic-rich substrates, corroborating evidence from fermented food systems where LAB mediate the biotransformation of dietary phenolics into bioactive metabolites (Li et al., 2021).

These results highlight the complex metabolic interplay between LAB and their *C. arabica* niches, indicating that adaptation to distinct coffee varieties drives functional specialization at the metabolomic level. Such specialization likely contributes not only to microbial ecological fitness but also to potential applications in food fermentation and probiotic functionality. In contrast, *Weissella* strains, characterized by organic acid and simple aromatic metabolite production, may enhance acidity, brightness, and overall cleanliness of the beverage profile.

From an applied perspective, strategic co-cultivation of selected lactobacilli and *Weissella* strains could balance aromatic depth with vibrant acidity, generating a more complex and consumer-preferred sensory profile. Future studies integrating sensory evaluation and volatile compound analysis will be essential to directly link metabolomic outputs with flavor perception. Such integrative studies could ultimately lead to the development of microbial consortia as functional bio-tools for customized fermentation and differentiated specialty coffee products.

### 3.6 Polyphenol and flavonoid variability in LAB strains

Total phenolic content (TPC, mg GAE/L) and flavonoids (TFC, mg QE/L) were measured in the CFS of the six LAB strains (B3, B6, B9, B10, B17, and B19). The findings revealed significant differences between the strains, suggesting a high degree of functional diversity in their ability to produce or accumulate bioactive metabolites with antioxidant functions. To reduce the interference of compounds from the culture medium, the extracts were obtained through liquid-liquid extraction with ethyl acetate, a method that excludes amino acids and major polar metabolites (Filannino et al., 2014). The analysis of the negative control (MRS without inoculation) showed a signal below the detection limit, confirming that the organic fraction predominantly contains metabolites generated by LAB, and not culture medium-derived compounds.

**FIGURE 8:**
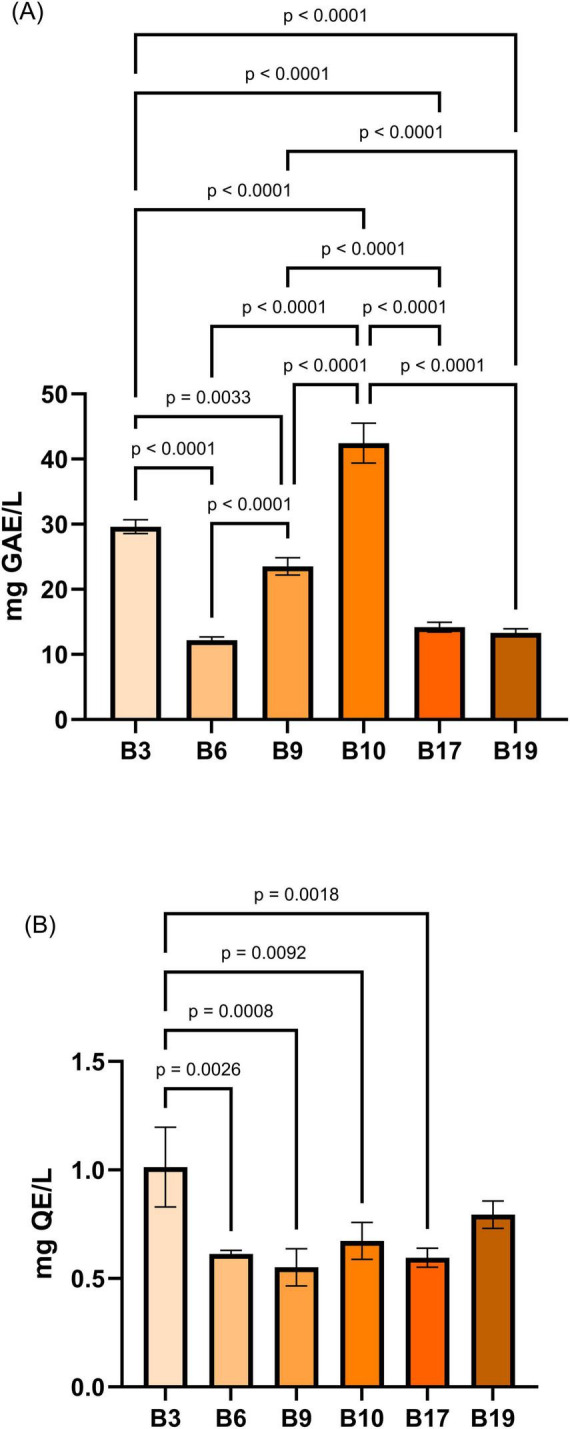
Total polyphenol and flavonoid content in cell-free supernatants of LAB isolates. **(A)** Polyphenols (mg gallic acid equivalents/L, mg GAE/L). **(B)** Flavonoids (mg quercetin equivalents/L, mg QE/L). Data are presented as mean ± SD (*n* = 3). Statistical differences among strains were determined by one-way ANOVA with *post-hoc* tests (*p*-values shown).

From Figure 8A, it can be observed that B10 and B3 exhibited the highest (42.44 ± 3.06 mg GAE/L and 29.61 ± 1.06, respectively), while B19 the lowest (13.32 ± 0.64 mg GAE/L) phenolic content. Multiple statistically significant differences have been recorded between almost all samples, especially, B10 and B3 are significantly richer in polyphenols compared to the other samples (*p* < 0.0001, *p* < 0.01).

In the case of the total flavonoid content (Figure 8B), sample B3 had the highest flavonoid content (1.013 ± 0.184 mg QE/L), significantly different from all the other samples (*p* < 0.01, *p* < 0.001, etc.). The rest of the samples (B6, B9, B10, B17, B19) had lower levels, ranging from 0.551 ± 0.085 mg QE/L (B9) to 0.794 ± 0.063 mg QE/L (B19), with no significant differences between them. The experimental data on the TPC and TFC met the conditions for applying the ANOVA test, as the Brown–Forsythe test indicated homogeneity of variances (*p* > 0.05), and the normality of the residuals was confirmed by the Shapiro–Wilk test, with *p*-values > 0.05 for both data sets.

The TPC and TFC results provide additional experimental support for the claim that LAB strains isolated from *C. arabica* cherries possess distinct metabolic signatures correlated with their phytochemical niches. Samples B3 and B10 demonstrate a superior capacity to biosynthesize or accumulate phenolic compounds and flavonoids. This is consistent with the previously observed enrichment in the metabolism of aromatic amino acids (biosynthesized by microorganisms or produced by the degradation of protein content in the MRS medium) and in the biosynthesis of compounds derived from tryptophan, which serve as precursors for volatile phenols and flavonoid derivatives (Okoye et al., 2023). The highest concentration of flavonoids and polyphenols was found in the CFS of strain B3, indicating that this sample could considerably stabilize volatile compounds, enhancing their aromatic complexity and endurance (Farag et al., 2022; Aloo et al., 2024). Its antioxidant properties could potentially be useful. CFS from strains B6, B9, B17, and B19 can maintain the acidity and freshness of coffee through alternative metabolic processes like the production of organic acid or peptides, even in the case of a decreased phenolic concentration.

It should be noted, however, that the TPC and TFC analyses were limited to the ethyl acetate-extractable fraction, which excludes polar metabolites and amino acids. Consequently, the results may not capture the full spectrum of bioactive compounds produced by the LAB strains, and additional analyses of the polar fraction would be necessary to obtain a more complete metabolic profile. Nevertheless, the observed variability among strains, with B3 and B10 exhibiting the highest levels of phenolic compounds (*p* < 0.0001), aligns with the previously observed enrichment of metabolic pathways and underscores the role of the plant substrate in shaping the functional and aromatic properties of LAB strains (da Silva Vale et al., 2024; Abbaspour, 2024).

## 4 Conclusion

This study demonstrates that LAB strains isolated from *Coffea arabica* cherries exhibit distinct, strain-specific metabolic signatures shaped by their ecological niche. *Lactiplantibacillus* strains were enriched in secondary metabolites such as harmala alkaloids and aromatic amino acid derivatives, contributing to fruity, floral, and sweet notes, while *Weissella confusa* showed higher levels of organic acids and simple aromatics, supporting acidity and brightness. FTIR analysis confirmed molecular changes in the fermentation matrix, including the release of extracellular polysaccharides, proteins, and phenolic compounds, with strain-specific spectral features suggesting functional contributions to texture, bioactivity, and flavor modulation. These findings highlight the dual role of *de novo* biosynthesis and enzymatic hydrolysis of complex substrates in shaping the extracellular metabolite pool, emphasizing enzymatic remodeling as a key mechanism in coffee fermentation. The study provides a foundation for leveraging the natural diversity of LAB strains to guide targeted fermentation strategies, enabling producers to balance aromatic richness, acidity, and freshness to enhance sensory quality and product differentiation. Future work should combine targeted metabolomics, genomic analyses, and sensory evaluation to confirm metabolite origins, elucidate enzymatic mechanisms, and directly link metabolomic profiles with flavor attributes. Such integrative approaches will support the development of next-generation microbial consortia for precision fermentation and high-value specialty coffee production.

## Data Availability

The original contributions presented in this study are included in this article/Supplementary material, further inquiries can be directed to the corresponding author.

## Footnotes

1 https://systemsomicslab.github.io/compms/msdial/main.html#MSP, accessed 15 April 2025

2 http://www.metaboanalyst.ca/, accessed on 22 April 2025

3 http://www.kegg.jp/, accessed on 20 May 2025
