# Supplementary material for: Metabolomic profiling of indigenous lactic acid bacteria reveals functional traits shaping the flavor and bioactivity of Ecuadorian coffee
Source: Front Microbiol. 2025 Nov 19;16:1697280. doi: 10.3389/fmicb.2025.1697280 (PMC12672526; doi:10.3389/fmicb.2025.1697280)
Supplement: Supplementary file 1 [file Supplementary_file_1.docx]

Supplementary Material

**Metabolomic Profiling of Indigenous Lactic Acid Bacteria Reveals Functional Traits Shaping the Flavor and Bioactivity of Ecuadorian Coffee**

Victor Cifuentes^1^, Ioana C. Marinas^2^, George Cătălin Marinescu ^3,4^, Roua Gabriela Popescu^3,4^, Mariana C. Chifiriuc ^2^, and Gabriela N. Tenea^1^*

^1^Biofood and Nutraceutics Research and Development Group, Faculty of Engineering in Agricultural and Environmental Sciences, Universidad Técnica del Norte, Ibarra 100150, Ecuador.

^2^Research Institute of the University of Bucharest-ICUB, University of Bucharest, 91-95 Splaiul Independentei St., 5050095 Bucharest, Romania

^3^Independent Research Association, 58 Timișului, Sector 1, 012416 Bucharest, Romania.

^4^Blue Screen SRL, 58 Timișului, Sector 1, 012416 Bucharest, Romania.

**Figure.S1**. Deconvolution vs. reference spectrum of some compounds detected by LC-MS analysis. (A). Harmane; (B) Daidzein; (C) Genistein; (D) Hexaethylene glycol; (E) FruLeuIle.

**Figure. S2**. Bubble plots depicting pathway impact analysis for B3 and B6. The y-axis indicates pathway enrichment significance (−log10 p-value), while the x-axis reflects pathway impact, representing the relative influence of detected metabolites within each pathway. Bubble size corresponds to pathway impact, and color gradient (red to yellow) denotes significance level, with red indicating the highest statistical significance

**Figure S3**. Bubble plots depicting pathway impact analysis for B9. The y-axis indicates pathway enrichment significance (−log10 p-value), while the x-axis reflects pathway impact, representing the relative influence of detected metabolites within each pathway. Bubble size corresponds to pathway impact, and color gradient (red to yellow) denotes significance level, with red indicating the highest statistical significance

**Figure S4.** Bubble plots depicting pathway impact analysis for B17. The y-axis indicates pathway enrichment significance (−log10 p-value), while the x-axis reflects pathway impact, representing the relative influence of detected metabolites within each pathway. Bubble size corresponds to pathway impact, and color gradient (red to yellow) denotes significance level, with red indicating the highest statistical significance
